# Effects of Maternal Undernutrition during Mid-Gestation on the Yield, Quality and Composition of Kid Meat Under an Extensive Management System

**DOI:** 10.3390/ani9040173

**Published:** 2019-04-17

**Authors:** Xiaoling Zhou, Qiongxian Yan, Hong Yang, Ao Ren, Zhiwei Kong, Shaoxun Tang, Xuefeng Han, Zhixiong He, Musibau Adungbe Bamikole, Zhiliang Tan

**Affiliations:** 1 CAS Key Laboratory for Agro-Ecological Processes in Subtropical Region, National Engineering Laboratory for Pollution Control and Waste Utilization in Livestock and Poultry Production, South-Central Experimental Station of Animal Nutrition and Feed Science in Ministry of Agriculture, Institute of Subtropical Agriculture, The Chinese Academy of Sciences, Changsha 410125, China; zxldky@126.com (X.Z.); 13723865920@139.com (H.Y.); renao606@163.com (A.R.); kongzhiwei2013@126.com (Z.K.); shaoxuntang@163.com (S.T.); xfhan@isa.ac.cn (X.H.); zxhe@isa.ac.cn (Z.H.); 2 University of The Chinese Academy of Science, Beijing 100049, China; 3 College of Animal Science, Tarim University, Alaer 843300, China; 4 Hunan Co-Innovation Center for Utilization of Botanical Functional Ingredients, Changsha 410128, China; 5 Hunan Co-Innovation Center of Animal Production Safety, Changsha 410128, China; 6 Department of Animal Science Faculty of Agriculture, University of Benin, Benin P.M.B.1154, Nigeria; bankymao@yahoo.co.uk

**Keywords:** maternal undernutrition, kid meat production, meat quality, fatty acid, amino acid

## Abstract

**Simple Summary:**

Nutrition status during pregnancy affects the meat production of offspring. In ruminants, the nutrient supply during the first and third periods of gestation is generally stressed, whereas the nutrition level during the second period of gestation is given less attention, in particular under the extensive husbandry system. This study focused on the effects of a 40% maternal undernutrition during mid-gestation, on the yield, quality, and composition of kid meat under an extensive system. The meat yield of the kids was decreased, while the meat quality and chemical composition, including the amino acid and fatty acid profiles, were unaffected. In meat production under an extensive husbandry system, the importance of the nutrient supply during mid-gestation in ruminants should be stressed.

**Abstract:**

Nutritional status during mid-gestation is often ignored under extensive husbandry. This study aimed to examine the effect of maternal undernutrition during mid-gestation on kid meat production under an extensive system. Twenty-seven goats (45 ± 3 d of gestation) were randomly assigned to an unrestricted group (100% of nutrient requirements), or a restricted group (60% of nutrient requirements from 45 to 100 d of gestation, and then re-alimented to 100%). Among the offspring, 16 eligible kids (eight per treatment) were selected, based on birth type and survival, and were harvested to evaluate the meat yield, quality, and composition at 90 d after birth. Maternal undernutrition reduced the body weight and size, average daily gain and hot carcass weight of the kids (*p* < 0.05). The lightness of the meat at 45 min postmortem was increased (*p* = 0.029) in the restricted kids. Apart from an increase in tyrosine concentration (*p* = 0.046), the proximate composition and the amino acid and fatty acid profiles were unaffected in the restricted kids (*p* > 0.05). Overall, maternal undernutrition during mid-gestation decreased the yield of kid meat, but did not significantly modify the quality and composition. These results highlight the importance of nutrient status during mid-gestation in the meat production of small ruminants under an extensive regime.

## 1. Introduction

Meat products from young livestock under an extensive system, such as veal, lamb and kid, compose a prosperous consumption category that is gaining higher demand from retailers and consumers, because of the low fat content, distinctive taste and good animal welfare associated with these products [1,2]. Unlike in an intensive system, young livestock under an extensive system encounter less digestive system abnormality during weaning stresses, and they are rarely confined to narrow spaces during short-term fattening. Therefore, in addition to the management and dietary factors that mainly affect meat production in intensive husbandry [1], the development of muscular tissue in utero, and during the early life after birth, determines the meat production of young livestock under an extensive system.

The myogenesis process originates from the differentiation of myogenic precursor cells from the paraxial mesoderm into myoblasts; the myoblasts then fuse into primary myofibrils during the first period of gestation. Next, secondary myofibrils are generated from progenitor cells, followed by further differentiation into mature myofibers during mid-gestation [3]. Unlike primary myofibrils, any harm to the development of secondary myofibrils during this stage cannot be reversed [4,5]. Thus, the development of secondary myofibrils during mid-gestation defines the number and composition of muscle fibers in later life to a large extent [6], and further determines the growth rate, muscle mass and meat quality of descendants after birth [7].

In extensive husbandry, the seasonal nutrient deficiency of the pregnant dams remains a harsh challenge worldwide. Much attention is focused on the nutrient supply at the stage of periconception and late gestation in the stockbreeding practice, but nutrient requirement during mid-gestation is generally neglected [8]. In particular, meat production of young livestock under this rearing system is more vulnerable to maternal undernutrition. Recently, the effect of maternal undernutrition during mid-gestation on muscle mass, meat quality and meat composition, has been evaluated in mature offspring after intensive fattening in several studies [9,10,11]. These observations revealed that meat mass and meat quality were partly damaged, but the alteration in meat composition was negligible. However, the impacts of maternal undernutrition during this stage under an extensive system on the meat production of young stock are important for research and practice, but remain unknown.

Thus, using meat-producing goats, the influences of maternal undernutrition during mid-gestation on the growth performance, meat yield, meat quality and meat composition, including amino acids and fatty acids, of kids under an extensive system, were examined.

## 2. Materials and Methods

### 2.1. Experimental Design and Animal Management

All experimental protocols were approved by the Animal Care Committee according to the Animal Care and the Use Guidelines of the Institute of Subtropical Agriculture at the Chinese Academy of Sciences (No. KYNEAAM-2013-0009).

The local meat-producing female goats of the *Xiangdong* black breed were naturally mated with the same buck, and grazed on a native shrub grassland in the subtropical region of China. After detection through portable ultrasonography (Aloka SSD-500 with a 5-MHz linear probe; Aloka, Shanghai, China), twenty-seven pregnant goats (45 ± 3 d of gestation) were randomly assigned to two groups, according to body weight (BW) and age: An unrestricted control group [UR group, 30.6 ± 12.0 kg BW, 4.1 ± 1.9 years, fed 100% of nutrient requirements according to the feeding standard of meat-producing sheep and goats (2004) [12], n = 12] and a restricted group (R group, 29.5 ± 8.5 kg BW, 4.1 ± 1.8 years, fed 60% of global nutrient requirements, n = 15). The nutrient limitation was performed by allocating a 60% ration allowance to the restricted dams to simulate a seasonal feed deficiency. 

To control the intake of dry matter (DM) and nutrients, dams were penned individually and fed twice daily (0800 and 1600 h) with a 50:50 ratio of concentrate to freshly mowed pasture grass from 45 to 100 d of gestation. The pasture grass mainly comprised of *Miscanthus*. All animals were free to drink water. The amounts of feed served to and refused by the dams in each pen were recorded daily. Feed samples were collected every 10 d for the analysis of DM (method no. 934.01) [13], crude protein (CP, method no. 2001.11) [13], ether extract (EE, method no. 2003.05) [13], Ca (method no. 927.02) [14], P (method no. 965.17) [14], and ash (method no. 942.05) [14], as well as acid detergent fiber (ADF) and neutral detergent fiber (NDF) according to Van Soest, et al. [15], with amylase and sodium sulfite included. The ingredients and composition of the diet for the dams are presented in Table 1. 

After feed restriction was withdrawn at 101 d of gestation, all dams were commingled and freely raised as a common group with the diet above fed *ad libitum*. During the period of feed restriction, one pregnant goat in the R group miscarried, while the other 26 dams gave birth naturally between 145 and 155 d of gestation. After kidding, each dam and her neonatal kids were penned together in one stall to prevent cross breastfeeding. The neonatal kids were naturally nursed until weaning. The same diet was provided *ad libitum* as before with the feed intake measured daily and allowance adjusted every week, in order to maintain the leftover feed was less than 10%.

The kidding performance of the dams is shown in Table 2. During 50 and 60 d after birth, the kids were preweaned by separation from their mothers during the daytime from 0800 to 1600 h and provided a fodder mixture of starter and freshly mowed *Miscanthus* grass at the ratio of 50:50. After complete weaning at 60 d, the kids were freely raised with the fresh *Miscanthus*, and the starter of 150 g/d was provided twice daily at 0800 and 1600 h with drinking water available. The amounts of feed served to and refused by the kids in each pen were recorded daily. The DM intake of the kids averaged 0.3 kg/d between 60 and 90 d. The ingredients and composition of the diet for the kids are presented in Table 1.

The cold and wet winter climate (the mean minimum temperature in the coldest month was 4.2 °C during the experiment period) of the research location (28.09 N to 28.25 N, 113.65 E to 113.91 E) increased the mortality rate of the neonatal kids, while the slotted ground of the barn and insufficient heat insulation measure (straw bedding) used in this study worsened this situation. Thus, the overall survival rate of kids at 90 d of age was low (Table 2). To eliminate the potential effect of the litter size and survival rate in each litter on the growth and development of kids between groups, the offspring were selectively harvested based on birth type and survival. Eight harvested kids in each treatment group were matched according to litter size and survival number in each litter, which both consisted of two singletons, two sets of twins (one set of twins with two surviving offspring and another set of twins with one surviving offspring), and two sets of triplets (one set with two surviving offspring and another set with one surviving offspring). The unmatched animals were not considered in the study. The average initial BW of the dams and types of litter size of these selected kids are shown in Table 2. The ratios of males to females among these selected kids were 0.60 and 1.00 in the UR and R groups, respectively.

### 2.2. Body Weight and Body Size Measurement

The body weight of the dams was measured before morning feeding at 45, 100 and 130 d of gestation and 60 d of weaning. The weight gain (kg) of the dams relative to the initial BW at 45 d of gestation was calculated. The BW, diagonal length (from the end of the shoulder to the point of the buttocks), height at the withers, heart girth (just behind the scapula), and cannon girth (at the middle of the cannon bone of the left forelimb) of the kids were measured immediately at birth and at 30, 60 and 90 d of age, respectively. The average daily gain (ADG, g/d) of the kids relative to the birth weight was calculated at 30, 60, and 90 d of age.

### 2.3. Slaughtering, Carcass Traits and Meat Quality Determination

After a 24-h feed withdrawal, the eight selected kids per group were weighed to obtain the fasting BW. Followed by electrical euthanasia and jugular exsanguination, the hot carcass weight was measured after removal of the skin, head, hoofs, tail, and visceral organs. The hot carcass yield was calculated as follows: hot carcass yield (%) = *hot carcass weight* (kg)/*fasting BW* (kg) × 100%. The left *Longissimus thoracis* (LT) was dissected, and the loin muscle (LM) area between the left 12th and 13th ribs was determined by the following equation: LM area = width × height × 0.7. Loin sections from the 10th to 12th ribs were collected for pH and meat color determination. The pH_45min_ (pH value at 45 min postmortem) and pH_24h_ (at 24 h postmortem) values were measured using a pH meter (205, Testo SE & Co, Freiburg, Germany), which was calibrated at the beginning of each measuring day using pH 4.6 and 7.0 buffers. The meat color was measured using a chroma meter (CR-400, Konica Minolta Group, Tokyo, Japan) that was calibrated against a standard white plate (8-mm-diameter aperture, d/0 illumination system) according to the Commission Internationale de I’Éclairage (CIE) L* (lightness), a* (redness), and b* (yellowness) system at 45 min (L*_45min_, a*_45min_ and b*_45min_) and 24 h (L*_24h_, a*_24h_ and b*_24h_) postmortem, as described by Alvarenga, et al. [16]. The average values of pH and color were determined after measuring three times at three locations on the ventral surface of the muscle. Approximately 5 g of LT muscle from the 8th to 10th ribs placed between 10 strata of Whatman No. 1 filter paper was pressed with a load of 35 kg for 5 min, and the percentage of water loss was obtained as the percentage of weight of released water relative to intact meat, as described by Li, et al. [17].

### 2.4. Chemical Composition, Amino Acid and Fatty Acid Determination

Other aliquots of the LT muscles from the 5th to 8th ribs were stored at −20 °C and then lyophilized for the analysis of meat composition, including moisture (method no. 934.01), protein (method no. 2001.11) and intramuscular fat content (method no. 2003.05), according to AOAC [14], as well as fatty acids and amino acids.

Fatty acids were analyzed through gas chromatography (7890A, Agilent Technologies, Santa Clara, CA, USA) with a flame ionization detector (FID) and SP-2560 column (100 m × 0.25 mm × 0.2 μm); the details of the procedures were described by Tan, et al. [18]. Fatty acid methyl esters were quantified by determining areas under the identified peaks. The results are expressed as a proportion of each fatty acid methyl ester in relation to the total methyl esters detected. Amino acids were measured with an automatic amino acid analyzer (L-8800, Hitachi Ltd., Tokyo, Japan) through liquid-phase hydrolysis according to Gilka, et al. [19]. The results are presented as the content of each amino acid in mg per 1 g of LT muscle (DM basis).

### 2.5. Statistical Analysis

All data were analyzed using IBM SPSS 19.0 statistical software (IBM Corp., Armonk, NY, USA). Data from the dams were analyzed using an independent-samples Student’s *t* test. The differences in growth performance in the kids at 30, 60, and 90 d after birth were examined by a repeated mixed model with treatment, age, sex and litter size as the fixed factors and the birth weight of the kids as the covariate. The estimation method was the Restricted Maximum Likelihood (REML). The data on the body weight and size at birth, and carcass and meat traits at 90 d after birth were analyzed by a mixed model with treatment, sex and litter size as the fixed factors and the initial BW of the dams as the covariate. Statistical significance was considered at *p* < 0.05, and all data are presented as the means and standard errors of the means (SEM).

## 3. Results

### 3.1. Kidding Performance, DM Intake, and BW of Dams

The birth rate and survival rate of the kids at 90 d after birth were unaffected (*p* > 0.05) by maternal undernutrition (Table 2). The DM intake, BW and weight gain of the dams are presented in Table 3. During the feed restriction period from 45 to 100 d of gestation, the DM intake of the dams in the R group was 32.3% lower (*p* = 0.014) than that in the UR group. The degree of global undernutrition in the R group was 67.7%, which was slightly lower than the 40% restriction level that we predesigned. During the lactation period from kidding to 60 d after parturition, the DM intake of dams was similar between the two groups (*p* = 0.298). Correspondingly, the weight gain during restriction decreased (*p* = 0.014) by 55.2% in the R group compared with that in the UR group. Nonetheless, the BW and weight gain of the dams during re-alimentation from 130 d of gestation to 60 d after parturition did not differ between the two groups (*p* > 0.05).

### 3.2. Body Weight and Body Size of Kids

Maternal undernutrition decreased the cannon girth of the kids at birth (*p* = 0.02), but the BW, diagonal length, height at the withers, and heart girth at birth were unaffected (*p* > 0.05). Maternal undernutrition decreased the BW, ADG, diagonal length, height at the withers, heart girth and cannon girth of the kids after birth (*p* < 0.01, Table 4). The BW and body size increased linearly with age (*p* < 0.01), whereas ADG decreased with age (*p* = 0.028). In addition, the height at the withers was affected by litter size (*p* = 0.025) and the cannon girth was affected by sex (*p* = 0.022).

### 3.3. Carcass Trait and Meat Quality of the Kids

The carcass traits and meat quality of the kids are presented in Table 5. Maternal undernutrition reduced the hot carcass weight (*p* = 0.017) of the R kids compared with that of the UR kids. The L*_45 min_ value in the R kids was increased by the treatment (*p* = 0.029), whereas the LM area, water loss percentage, pH_45min_, pH_24h_, a*_45 min_, b*_45 min_, L*_24 h_, a*_24 h_, b*_24 h_, protein content, intramuscular fat content and moisture content were unaffected (*p* > 0.05). In contrast to the significant effect of the litter size on b*_24 h_ (*p* = 0.008), sex and litter size did not influence carcass traits and meat quality (*p* > 0.05).

### 3.4. Fatty Acids and Amino Acids in the LT Muscle of the Kids

The fatty acid profile and amino acid concentration of LT in the kids are presented in Table 6 and Table 7, respectively. Maternal undernutrition did not alter the fatty acid profile, including the medium- and long-chain fatty acids of the LT muscle (*p* > 0.05). However, C18:0 was affected by sex (*p* = 0.001) and litter size (*p* = 0.030), whereas SFA, UFA and UFA/SFA were affected by sex (*p* < 0.01). The concentration of tyrosine in the LT muscle in the R kids was increased compared with that in the UR kids (*p* = 0.046), whereas the remaining 15 amino acids were unaffected by treatment, sex and litter size (*p* > 0.05).

## 4. Discussion

In extensive production systems, the shortage of roughage, especially high-quality forage in ruminants, is a common problem in the cold season of China [20], leading to low intake and overall nutrient deficiencies. Severe malnutrition during pregnancy (higher than 50%) often leads to pregnancy failure, while the effects of moderate (30 to 40%) or mild (less than 20%) undernutrition are often unnoticed. By modeling a moderate intake restriction (32.3%) under an extensive rearing system, this study aimed to examine the impact of overall maternal undernutrition during mid-gestation on the meat production of young offspring. We found that maternal nutrition deficiency during mid-gestation exerted a more severe effect on meat yield (ADG and carcass weight) of kids than that on meat quality and composition.

The nutritional status during gestation is reflected as an alteration in the BW of the pregnant dams; meanwhile, the nutrient supply from the mother via umbilical cord circulation is the exclusive nutritional source for the fetuses [21]. In this study, nutrient restriction by 1/3 of the maintenance requirement during mid-gestation reduced the weight gain of pregnant dams during restriction, and the BW, ADG and body size in the restricted kids after birth were reduced, while the BW and body size of the restricted kids at birth were not significantly affected. However, after exposure to maternal undernutrition during mid-gestation, Piaggio, et al. [11] reported a lower birth weight of the lambs but no difference in later BW under a 70% metabolizable energy restriction. In contrast, Sen, et al. [9] reported no effect on the birth and weaning weights of lambs under 50% nutrition restriction, and McGregor [22] did not observe a difference in the birth weight of kids suffering from maternal undernutrition during this stage. In this study, maternal undernutrition had no effect on birth weight but reduced the growth of the offspring after birth. The reason for this discrepancy in the growth of offspring at birth and after birth is partly related to the restriction level of the dams. Moreover, as proposed by Piaggio, et al. [11], the refeeding status of the mother during later gestation and the probability that the offspring can compensate for the in utero retardation after birth are associated with this discrepancy. Although the BW of the restricted dams was restored during late gestation and lactation after refeeding (38.5 kg in UR dams versus 36.6 kg in R dams at 130 d of gestation, and 31.4 kg in UR dams versus 32.8 kg in R dams at weaning) in this study, the 67.7% mid-gestational undernutrition still exerted a severe growth retardation effect on the postnatal kids. In contrast to the dams, compensation growth of the kids did not occur during the first three months in this study. This result suggests that the growth lag of kids may be mediated by other factors, such as maternal mammary gland development or milk production. This is because breast milk is the main nutrient source for the neonatal offspring in the extensive rearing system, and mammary gland development, milk yield and composition in sheep are altered by undernutrition during mid- and late-gestation [23,24]. Since milk yield and composition were not measured in this study, the actual effect of maternal mammary gland development and milk production on the growth of kids could not be determined. However, the maternal undernutrition during mid-gestation clearly impeded the growth of the offspring in the extensive feeding system.

Maternal nutrition during mid-gestation has profound effects on muscle development and meat production in ruminants [7]. In this study, the hot carcass weight in the restricted kids at 90 d of age was decreased. Similarly, Sen, et al. [9] reported that 50% maternal undernutrition during mid-gestation decreased the hot carcass weight of five-month-old lambs. In contrast, no change occurred in the carcass weight of offspring when an 80% restriction level was applied to lambs [25] or beef cattle [26]. The impacts of maternal undernutrition during mid-gestation on meat yield are not consistent between these studies, likely due to differences in the maternal nutritional level, postweaning diet and management regime [1,2]. However, the 67.7% maternal undernutrition during mid-gestation decreased the meat production of kids in this extensive rearing system; thus, the importance of the nutrient requirement during mid-gestation in meat production under a similar production regime needs to be given more attention.

Compared with lamb meat, kid meat has the traits of a higher pH, more moisture, less intramuscular fat content, and a lighter color [2]. In this study, the lightness of fresh meat from the restricted kids was increased (L*_45 min_). This may hurt the market value in some countries, such as those in the Far-East regions, because their consumers prefer bright-red fresh meat without cooling and flushing. Meat color is mainly determined by myoglobin concentration, which has the greatest effect on meat lightness [27]. Lightness is negatively related to the myoglobin concentration, and a reduction in muscle myoglobin concentration is achieved by decreasing the proportion of oxidative muscle fibers, which has been observed in lambs exposed to maternal undernutrition during mid-gestation [9,28,29]. Thus, undernutrition during mid-gestation possibly led to an increase in L*_45 min_ via influencing the ratio of oxidative to glycolytic muscle fibers in kids. In addition, the greater L*_45 min_ and numerically greater water loss percentage in the restricted kids shortly after postmortem may raise a concern about the risk of Pale Soft Exudative Meat (PSE). PSE meat is characterized by a soft texture, light color, and high water loss percentage. The reason is the low pH caused by excessive glycolysis and accumulation of lactate in raw meat [30]. Nevertheless, the pH value at 45 min and 24 h post-mortem did not decrease; thus, this meat could not be deemed as PSE meat. The factors affecting the water-holding capacity of fresh meat are complex and have been extensively reviewed [31], and they include physiological, rearing, slaughtering and processing factors. In association with the influence on the meat color, the greater water loss is also possibly associated with the changes in muscle fiber composition, as muscles with a high percentage of oxidative fibers are assumed to have a better water-holding capacity than glycolytic muscles [30,31].

The proximate composition, amino acids and fatty acids are the flavor-forming ingredients that influence the palatability and flavor of meat. Proximate composition, including the moisture, protein and intramuscular fat content of the LT muscles in the kids was not affected by intake restriction in this study, consistent with the observations of Sen, et al. [9] in lambs. However, pertinent studies on the effects of maternal nutrition during mid-gestation on the amino acid or fatty acid profile of meat composition are lacking. Alvarenga, et al. [16] demonstrated that maternal nutrition restriction during periconception did not affect the fatty acid composition of beef in male progeny. In the present study, nutrient deficiency during mid-gestation also did not alter the fatty acid profile of kid meat. This is because the fatty acid profile of meat is mainly regulated by dietary factors [32,33], and the same diet was fed to the kids after weaning. Furthermore, the effects of maternal malnutrition on the amino acid concentration of the LT muscle were not significant except for the enhanced tyrosine in the restricted offspring. The reason is that the amino acid profiles are hardly influenced by animal production factors, including nutritional level and dietary composition [34].

## 5. Conclusions 

This study showed that moderate nutritional deficiency (nearly 1/3) during mid-gestation led to growth and developmental retardation of the offspring, which in turn reduced the carcass output. However, the meat quality and composition, including the amino acid and fatty acid profiles, of the meat were hardly affected. For the sustainable development of environmentally friendly and nutrition-favorable kid meat production, more attention should be paid to the feeding and management strategy of pregnant dams during the second period of gestation.

## Figures and Tables

**Table 1:** Ingredients and composition of the experimental diets for dams and kids (DM basis).

Item	Dams	Kids
Ingredients (%)		
*Miscanthus*	50.00	20.00
Maize	33.50	36.00
Wheat bran	-	14.40
Soybean meal	10.33	14.16
Whey powder	-	6.40
Fat powder	4.00	6.40
Calcium carbonate	0.49	0.24
Calcium hydrophosphate	0.46	0.80
Sodium chloride	0.22	0.40
Premix ^1^	1.00	1.20
Total	100	100
Composition		
Metabolic energy (MJ/kg) ^2^	11.78	15.19
Crude protein (%)	12.05	15.52
Ether extract (%)	8.97	10.46
Neutral detergent fiber (%)	64.44	24.99
Acid detergent fiber (%)	28.32	11.67
Ash (%)	5.89	3.59
Calcium (%)	0.53	0.76
Phosphorus (%)	0.20	0.32

^1^ Contained per kg of diet: 1.19 g MgSO_4_•H_2_O, 25 mg FeSO_4_•7H_2_O, 8 mg CuSO_4_•5H_2_O, 30 mg MnSO_4_•H_2_O, 50 mg ZnSO_4_•H_2_O, 0.1 mg Na_2_SeO_3_, 0.4 mg KI, 0.3 mg CoCl_2_•6H_2_O, 950 IU vitamin A, 175 IU vitamin D, and 180 IU vitamin E. ^2^ ME was calculated according to the data of Zhang [12].

**Table 2:** Kidding performance of dams and selection criterion for harvesting of kids.

	Treatment ^1^			
Item	UR	R	SEM ^2^	*p*-Value
n Kidding performance of dams	12	14	-	-
Birth rate (%)	175	160	23.19	0.651
Survival rate of kids at 90 d after birth (%)	68.1	53.6	11.27	0.375
Selection criterion for harvesting of kids				
Initial BW of dams (kg) giving birth to the selected kids	29.9	29.2	4.55	0.925
Number of selected kids in different types of litter size	8	8	-	-
From singletons (n)	2	2	-	-
From twins with two surviving kids (n)	2	2	-	-
From twins with one surviving kid (n)	1	1		
From triplets with two surviving kids (n)	2	2		
From triplets with one surviving kid (n)	1	1	-	-

^1^ Pregnant goats in the UR group were fed 100% of their nutrient requirements in reference to Chinese meat goat requirements (2004) [12]. Pregnant goats in the R group were fed 60% of the intake of the UR group during 45 to 100 d of gestation and then re-alimented to 100% of the nutrient requirements. ^2^ SEM = standard error of the mean.

**Table 3:** Effects of undernutrition during mid-gestation on DM intake, body weight and weight gain of dams.

	Treatment ^1^			
Item (kg)	UR	R	SEM ^2^	*p*-Value
n	6	6	-	-
Daily DM intake during 45 to 100 d of gestation	0.93	0.65	0.06	0.014
Daily DM intake after kidding	1.06	1.21	0.23	0.298
Weight at 100 d of gestation	35.8	31.9	5.02	0.596
Weight gain at 100 d of gestation ^3^	6.9	2.6	0.92	0.014
Weight at 130 d of gestation	38.5	36.6	5.57	0.809
Weight gain at 130 d of gestation ^3^	8.7	7.3	1.25	0.472
Weight at weaning	31.4	32.8	3.58	0.794
Weight gain at weaning ^3^	1.5	3.5	2.21	0.542

^1^ Pregnant goats in the UR group were fed 100% of their nutrient requirements in reference to Chinese meat goat requirements (2004) [12]. Pregnant goats in the R group were fed 60% of the intake of the UR group during 45 to 100 d of gestation and then re-alimented to 100% of the nutrient requirements. ^2^ SEM = standard error of the mean. ^3^ Weight gain was relative to the initial body weight of the dam at 45 d of gestation.

**Table 4:** Effects of maternal undernutrition during mid-gestation on the growth performance of the kids.

		Age				*p*-Values after Birth			
Item	Treatment ^1^	Birth	30 d	60 d	90 d	Treatment	Sex	Litter Size	Age
Body weight (kg)	UR	2.1	5.3	7.8	9.5	<0.001	0.849	0.405	<0.001
	R	1.8	4.0	6.0	7.8				
	SEM ^2^	0.14	0.17	0.18	0.51				
Average daily gain (g/d)	UR	-	104.2	92.6	81.6	0.001	0.543	0.853	0.023
	R	-	72.5	71.1	66.0				
	SEM	-	5.71	6.88	13.31				
Diagonal length (cm)	UR	24.8	34.0	40.8	44.9	<0.001	0.191	0.148	<0.001
	R	24.0	31.8	37.6	40.6				
	SEM	0.61	0.61	0.67	0.56				
Height at the withers (cm)	UR	27.1	34.0	38.7	41.9	<0.001	0.274	0.025	<0.001
	R	24.6	31.3	35.3	38.1				
	SEM	0.83	0.45	0.55	0.59				
Heart girth (cm)	UR	31.6	41.9	47.2	49.2	<0.001	0.979	0.233	<0.001
	R	29.1	37.2	43.9	45.1				
	SEM	0.96	0.80	0.80	1.07				
Cannon girth (cm)	UR	5.3	5.9	6.3	6.5	<0.001	0.022	0.056	0.001
	R	4.8	5.4	5.7	5.9				
	SEM	0.13	0.12	0.13	0.15				

^1^ Pregnant goats in the UR group were fed 100% of their nutrient requirements in reference to Chinese meat goat requirements (2004) [12]. Pregnant goats in the R group were fed 60% of the intake of the UR group during 45 to 100 d of gestation and then re-alimented to 100% of the nutrient requirements. ^2^ SEM = standard error of the mean.

**Table 5:** Effects of maternal undernutrition during mid-gestation on carcass traits and meat quality in the *Longissimus thoracis* of kids at 90 d of age.

	Treatment ^1^			*p*-Values		
Item	UR	R	SEM ^2^	Treatment	Sex	Litter Size
Hot carcass weight (kg) ^3^	3.78	2.78	0.24	0.017	0.461	0.773
Hot carcass yield (%)	42.10	39.09	1.12	0.115	0.881	0.805
LM area (cm^2^)	5.39	5.35	0.66	0.996	0.767	0.262
Water loss percentage (%)	9.73	11.37	1.24	0.431	0.606	0.474
pH45min ^4^	6.38	6.32	0.09	0.639	0.866	0.585
pH24h ^4^	5.61	5.63	0.05	0.375	0.103	0.490
L*45min ^4^	43.32	46.41	0.93	0.029	0.472	0.379
a*45min ^4^	12.46	10.78	0.95	0.258	0.371	0.589
b*45min ^4^	4.54	4.90	0.29	0.335	0.673	0.234
L*24h ^4^	48.25	51.78	1.66	0.387	0.441	0.359
a*24h ^4^	13.12	11.74	0.65	0.228	0.219	0.822
b*24h ^4^	6.68	7.97	0.64	0.115	0.146	0.008
Moisture (%)	77.67	78.53	0.66	0.426	0.870	0.974
Protein (%, DM basis)	76.06	76.53	0.81	0.602	0.334	0.329
Intramuscular fat (%, DM basis)	6.96	5.58	0.98	0.273	0.915	0.589

^1^ Pregnant goats in the UR group were fed 100% of their nutrient requirements in reference to Chinese meat goat requirements (2004) [12]. Pregnant goats in the R group were fed 60% of the intake of the UR group during 45 to 100 d of gestation and then re-alimented to 100% of the nutrient requirements. ^2^ SEM = standard error of the mean. ^3^ Carcass weight was the weight after removal of the skin, head, hoofs, tail and visceral organs after complete exsanguination. ^4^ Parameters measured at 45 min or 24 h postmortem. L*, a* and b* indicate lightness, redness, and yellowness, respectively.

**Table 6:** Effects of maternal undernutrition during mid-gestation on the fatty acid profile in the *Longissimus thoracis* of the kids at 90 d of age.

	Treatment ^1^			*p*-Values		
Fatty Acid Profile (% of Total Fatty Acids)	UR	R	SEM ^2^	Treatment	Sex	Litter Size
C14:0	3.2	2.7	0.43	0.419	0.998	0.315
C16:0	19.7	19.4	0.59	0.634	0.960	0.275
C16:1	2.0	1.7	0.19	0.254	0.171	0.266
C17:0	0.9	0.9	0.07	0.912	0.050	0.978
C18:0	16.4	18.3	1.03	0.106	0.001	0.030
C18:1 n-9, trans	3.0	2.6	0.21	0.091	0.083	0.768
C18:1 n-9, cis	33.5	31.4	1.81	0.480	0.497	0.191
C18:2 n-6	9.3	9.6	0.76	0.647	0.260	0.246
C20:0	0.1	0.1	0.01	0.115	0.080	0.256
C18:3 n-6	0.1	0.1	0.01	0.493	0.722	0.277
C18:3 n-3	0.1	0.1	0.01	0.840	0.741	0.293
C20:1 n-9	0.4	0.4	0.02	0.484	0.548	0.057
C20:3 n-6	0.5	0.6	0.08	0.617	0.709	0.405
C20:4 n-6	10.2	11.4	1.68	0.572	0.614	0.425
C22:6 n-3	0.5	0.5	0.06	0.771	0.487	0.362
SFA ^3^	40.3	41.5	1.14	0.621	0.007	0.993
UFA ^3^	59.7	58.4	1.11	0.519	0.007	0.947
UFA/SFA	1.5	1.4	0.06	0.443	0.006	0.939

^1^ Pregnant goats in the UR group were fed 100% of their nutrient requirements in reference to Chinese meat goat requirements (2004) [12]. Pregnant goats in the R group were fed 60% of the intake of the UR group during 45 to 100 d of gestation and then re-alimented to 100% of the nutrient requirements. ^2^ SEM = standard error of the mean. ^3^ UFA is the sum of C16:1, C18:1, C18:2, C18:3, C20:1, C20:3, C20:4 and C22:6. SFA is the sum of the remaining fatty acids.

**Table 7:** Effect of maternal undernutrition during mid-gestation on the amino acid concentrations in the *Longissimus thoracis* of kids at 90 d of age.

	Treatment ^1^			*p*-Values		
Concentration of Amino Acids (mg/g, DM Basis)	UR	R	SEM ^2^	Treatment	Sex	Litter Size
Asp	87.9	86.5	1.91	0.656	0.267	0.394
Thr	43.1	42.8	0.90	0.923	0.301	0.686
Ser	34.1	34.2	0.71	0.825	0.180	0.646
Glu	114.1	117.2	2.29	0.331	0.287	0.743
Gly	44.4	47.3	2.76	0.353	0.116	0.344
Ala	56.3	55.6	1.46	0.803	0.068	0.270
Cys	8.6	7.9	1.47	0.651	0.339	0.709
Val	45.5	45.4	1.06	0.943	0.452	0.550
Met	18.5	20.8	1.41	0.159	0.055	0.311
Ile	41.0	40.5	1.04	0.868	0.514	0.968
Leu	64.1	66.1	1.44	0.349	0.543	0.939
Tyr	22.1	26.7	1.35	0.046	0.714	0.644
Phe	29.0	30.2	0.96	0.376	0.572	0.753
Lys	82.4	80.8	1.96	0.589	0.468	0.536
His	28.0	26.5	0.88	0.347	0.092	0.769
Arg	56.7	59.2	1.75	0.283	0.194	0.752
EAA ^3^	416.8	420.2	7.09	0.782	0.309	0.583
NEAA ^4^	358.9	367.4	6.29	0.334	0.083	0.357
EAA/NEAA	1.2	1.1	0.01	0.350	0.455	0.323

^1^ Pregnant goats in the UR group were fed 100% of their nutrient requirements in reference to Chinese meat goat requirements (2004) [12]. Pregnant goats in the R group were fed 60% of the intake of the UR group during 45 to 100 d of gestation and then re-alimented to 100% of the nutrient requirements. ^2^ SEM = standard error of the mean. ^3^ EAA refers to essential amino acids and conditionally essential amino acids, including Thr, Cys, Val, Met, Ile, Leu, Phe, Lys, His and Arg. ^4^ NEAA refers to nonessential amino acids, including Asp, Ser, Glu, Gly, Ala and Tyr.
